# Emotional Regulation and Physiological Responses to a Cultural Heritage Virtual Reality Experience in Older Adults: Mixed Methods Study

**DOI:** 10.2196/91772

**Published:** 2026-07-14

**Authors:** Yafeng Lai, Pohsun Wang

**Affiliations:** 1 College of Fine Arts and Design, Guangdong Vocational Academy of Art, Guangzhou, China; 2 Faculty of Innovation and Design, City University of Macau, Avenida Padre Tomás Pereira, Taipa, Macau, China, 853 63180944

**Keywords:** digital therapy, cultural landscape, electrodermal activity, older adults, emotional health, Historic Centre of Macao

## Abstract

**Background:**

Against the backdrop of global population aging, the development of low-burden and widely acceptable interventions to support emotional health in older adults has become a critical research priority. Virtual reality (VR) has been increasingly applied in medical rehabilitation and psychological interventions due to its immersive and interactive features. However, its potential as a form of digital therapy that integrates cultural content and artistic design remains underexplored, particularly with respect to the mechanisms through which such experiences facilitate emotional regulation in older populations. Empirical studies combining subjective emotional outcomes with objective physiological indicators are especially scarce.

**Objective:**

This study aimed to examine the emotional regulation effects of an artistic cultural heritage–based VR experience among older adults and to explore its underlying mechanisms by integrating subjective psychological assessments with objective physiological responses.

**Methods:**

A mixed methods experimental design was adopted using the United Nations Educational, Scientific and Cultural Organization Historic Centre of Macao as the study context. Two VR conditions were developed for comparison: (1) an artistic cultural heritage VR environment and (2) a conventional photorealistic replica VR environment. A total of 49 participants aged 60 years and older were recruited. During the VR experience, electrodermal activity (EDA) was continuously recorded to capture emotional arousal. Physiological data were triangulated with pre- and postintervention psychological questionnaires assessing emotional states, as well as semistructured interviews.

**Results:**

Compared with the realistic replication VR condition, the cultural heritage VR condition was associated with significantly higher positive affect and lower negative affect (*P*<.05). At the physiological level, a significant between-group difference was observed in mean normal-to-normal interval (*P*=.01; Cohen *d*=0.91), whereas no significant differences were found in EDA measures (all *P*>.05). EDA results showed notable interindividual variability but did not demonstrate consistent group-level effects. These findings suggest that emotional changes may be more prominently reflected in subjective experience than in physiological arousal.

**Conclusions:**

By integrating subjective and physiological measures, this study provides preliminary empirical evidence that cultural heritage VR is associated with improved emotional outcomes in older adults. The findings propose a conceptual interpretative pathway linking digital cultural experience, emotional engagement, and psychological well-being, while highlighting the importance of individual variability in physiological responses. This study contributes to the development of content- and experience-oriented approaches in digital therapeutics and provides an evidence-based framework for emotion-supportive design in digital heritage and aging-related contexts.

## Introduction

The World Health Organization [1] projects that the global population aged 60 years and older will reach 2.1 billion by 2050. Mental health conditions such as depression and anxiety are expected to exert an increasingly significant impact on quality of life in later adulthood [2]. As population aging accelerates worldwide, enhancing emotional well-being and life satisfaction among older adults has become a central concern across public health, psychology, and design-related disciplines [3]. Declines in physical capacity and social participation increase vulnerability to loneliness, depression, and anxiety in later life, which in turn affect daily functioning and overall physical and mental health [2,4].

Traditional approaches to emotional regulation in older adults—such as art therapy, group activities, and cultural visits—are often constrained by mobility limitations, health conditions, or external disruptions such as pandemics [5,6]. In response, digitally mediated therapeutic interventions have emerged as a promising alternative [7]. Identifying effective digital strategies to stimulate positive emotions and psychological resilience in older populations has thus become a key focus of interdisciplinary research [8]. In this context, virtual reality (VR), owing to its immersive, controllable, and spatially representational properties [9], offers new opportunities to enhance emotional experiences among older adults [10-12].

Presence, commonly defined as the subjective sensation of “being there” within a mediated environment, represents a central psychological construct in VR research. A strong sense of presence can enhance users’ emotional engagement, attentional focus, and cognitive involvement in virtual environments. Previous studies have demonstrated that higher levels of presence are associated with stronger affective responses and more pronounced physiological reactions during immersive experiences. In particular, presence has been identified as an important psychological pathway through which immersive VR environments influence emotional and behavioral outcomes [13,14]. For example, the National Arts Council of Singapore, in collaboration with the National Gallery Singapore, has provided 1-hour virtual heritage art tours for older adults [15]. Although these programs have been widely welcomed and in high demand, empirical evaluations of their emotional impact remain limited.

Existing studies have demonstrated that VR-based interventions can alleviate depressive and anxiety symptoms and improve overall psychological well-being among older adults [16-18]. However, most prior research has focused on natural environments, cognitive training, or reminiscence-based content, with relatively little attention paid to immersive experiences that incorporate cultural depth and artistic expression [19-21]. Evidence suggests that older users—including individuals with dementia—prefer VR content related to travel, nature, art, social interaction, or familiar environments [22,23].

Unlike conventional cultural reproduction, cultural heritage–based VR emphasizes aesthetic transformation and contextual storytelling. It integrates historical spaces, cultural narratives, and artistic expression into immersive virtual experiences, rather than merely replicating physical environments. Such experiences may strengthen cultural identity and emotional immersion while eliciting deeper affective engagement. Despite this potential, heritage-based VR as a form of digital therapy remains underexplored at the intersection of VR research and aging studies.

Another limitation of this research lies in its heavy reliance on self-report measures, such as questionnaires and interviews, when examining the emotional effects of VR-based cultural experiences [8,24]. Objective physiological indicators are rarely incorporated. Electrodermal activity (EDA), a sensitive measure of autonomic nervous system activity, provides real-time information on emotional arousal and attentional engagement during immersive experiences [25]. Wearable physiological sensors have become an increasingly reliable method for collecting autonomic nervous system signals in naturalistic and experimental contexts [26]. When combined with self-report measures, EDA can reduce biases associated with retrospection and offer a dynamic representation of emotional fluctuations over time. Recent findings highlight the particular sensitivity of EDA responses to emotional arousal and presence in VR environments, underscoring its methodological value for emotional health research [27]. Incorporating EDA into VR-based cultural experience studies therefore represents an important methodological advancement.

To address these gaps, this study selected the Historic Centre of Macao as the experimental context, with a focus on the Ruins of St. Paul’s and its surrounding urban spaces. Prior research suggests that historical heritage settings can foster meaning, identity, and subjective well-being among older adults [28,29]. As a United Nations Educational, Scientific and Cultural Organization World Heritage site, Macao embodies distinctive historical narratives and artistic symbolism. The virtual environments were modeled according to real spatial proportions and architectural characteristics to recreate the physical form, spatial scale, and ambient qualities of the historic district. Building on this foundation, an “artistic cultural experience” design approach was introduced, embedding cultural information through aesthetic and interactive elements.

Through heritage-oriented VR experiences, VR was positioned not merely as a tool for spatial replication, but as a medium for artistic translation and emotional regulation [30]. From the perspective of digital cultural heritage and art experience research, artistic interpretation can intensify emotional engagement and perceived meaning, enabling virtual environments to function as affective and interpretive media rather than purely technical simulations [31,32]. By integrating EDA with subjective assessments, this study further explores the relationship between virtual cultural-artistic experiences and physiological emotional responses.

The contributions of this study are 3-fold. First, it advances VR content design from literal replication toward artistic reinterpretation, encouraging emotional resonance and identity engagement among older participants. Second, it combines EDA with subjective measures to examine whether a coordinated pathway exists linking experience, emotional arousal, and emotional health. Third, by using the Historic Centre of Macao as a case study, it explores the social value of cultural heritage digitization in health promotion contexts. This interdisciplinary approach provides both a novel empirical pathway and a model for integrating design research, environmental psychology, and aging studies. Theoretically, it elucidates how design-led content and physiological feedback jointly shape emotional mechanisms in later life. Practically, it offers a replicable framework for digital cultural experiences applicable to cultural institutions, community care settings, and urban cultural policy.

Based on the theoretical framework and the identified research gaps, this study aims to examine whether cultural heritage VR experiences can enhance emotional health among older adults and to explore the emotional mechanisms associated with such experiences. Specifically, this study addresses 3 core research questions (RQs): (RQ1) whether cultural heritage VR is more effective than realistic replication VR in improving emotional health among older adults, (RQ2) whether cultural heritage VR elicits stronger electrodermal responses reflecting emotional arousal, and (RQ3) whether individual psychological traits including emotion regulation difficulty and psychological resilience are associated with subjective emotional states and depression levels before and after VR intervention. Accordingly, 3 hypotheses (H1-H3) were formulated corresponding to these RQs (Table 1).

**Table 1.** Correspondence between research hypotheses (H) and research questions (RQs).

Research hypotheses	Corresponding RQs
H1. Older adults who experience cultural heritage VR^a^ will exhibit higher levels of emotional health than those in the realistic replication VR condition.	RQ1. Can digital therapeutics based on cultural heritage VR significantly improve emotional health in older adults?
H2. Cultural heritage VR experiences will elicit stronger electrodermal responses, reflecting greater emotional arousal.	RQ2. Does cultural heritage VR induce significantly higher electrodermal activity, indicating stronger arousal, compared with the control condition?
H3. Individual psychological traits such as difficulty in emotion regulation and psychological resilience are significantly correlated with emotional states (PA^b^/NA^c^) before and after VR intervention.	RQ3. Do individual psychological traits such as emotion regulation ability and psychological resilience influence the intervention effect of VR cultural heritage experience on the emotional health of older people?

a VR: virtual reality.

b PA: positive affect.

c NA: negative affect.

## Methods

### Ethical Considerations

Older adult participants were recruited through local community committees and care institutions for older people in Zhuhai and Foshan, Guangdong Province. All participants met the study inclusion criteria and participated on a voluntary basis. Ethics approval for this study was granted by the Academic Committee of the School of Innovative Design, City University of Macao (approval Ref 2025 09291315). Written informed consent was obtained from all participants prior to study initiation. Participants’ capacity to provide informed consent was independently assessed by a licensed clinical psychologist who was not involved in the study, in order to ensure adequate comprehension and voluntary participation. All collected data were anonymized. Data handling and storage complied with the Organic Law on the Protection of Personal Data and Guarantee of Digital Rights (Law 3/2018, enacted on December 5, 2018). The study was conducted in accordance with good research practice standards and the principles of the Declaration of Helsinki.

### Participants

The sample size was estimated based on effect sizes reported in previous studies examining the impact of VR interventions on physiological outcomes, including heart rate variability (HRV) and EDA. Prior studies have demonstrated that VR-based interventions can significantly modulate autonomic and physiological responses, suggesting moderate to large effects [33,34]. Based on these estimates, a minimum of 30 participants per group was required to achieve 80% statistical power at a significance level of .05.

To further account for potential attrition during the VR intervention and follow-up assessments, an anticipated dropout rate of approximately 18% was incorporated, based on prior research involving older adults in VR settings [35]. Accordingly, the target sample size was increased, resulting in a final sample of 49 older adults; the study aimed to recruit at least 30 participants per group. A final sample of 49 older adults was included in the analysis.

Participants were eligible for inclusion if they met the following criteria: (1) aged 60 years or older and able to complete the experimental procedures independently or with assistance; (2) able to understand the study procedures and provide written informed consent, with additional auditory or visual explanations provided when necessary; (3) no uncontrolled cardiovascular disease or epilepsy (participants with stable conditions were included only after clinical approval); and (4) no severe cognitive impairment. Cognitive status was screened using the Mini-Mental State Examination, with a cutoff score of ≥24 indicating eligibility.

Exclusion criteria were (1) a history of severe motion sickness, recent ear surgery, or dermatological conditions that could interfere with EDA electrode attachment; and (2) recent use of medications known to strongly affect autonomic nervous system activity. When medication use could not be avoided, it was recorded and treated as a covariate in subsequent analyses.

This randomized experiment had a total duration of 3 months and concluded on November 30, 2025. Participant recruitment was conducted between September and November 2025. The baseline assessment took place on September 1, 2025, and the final assessment was completed on November 30, 2025.

### Research Materials

#### Overview

**Figure 1.**
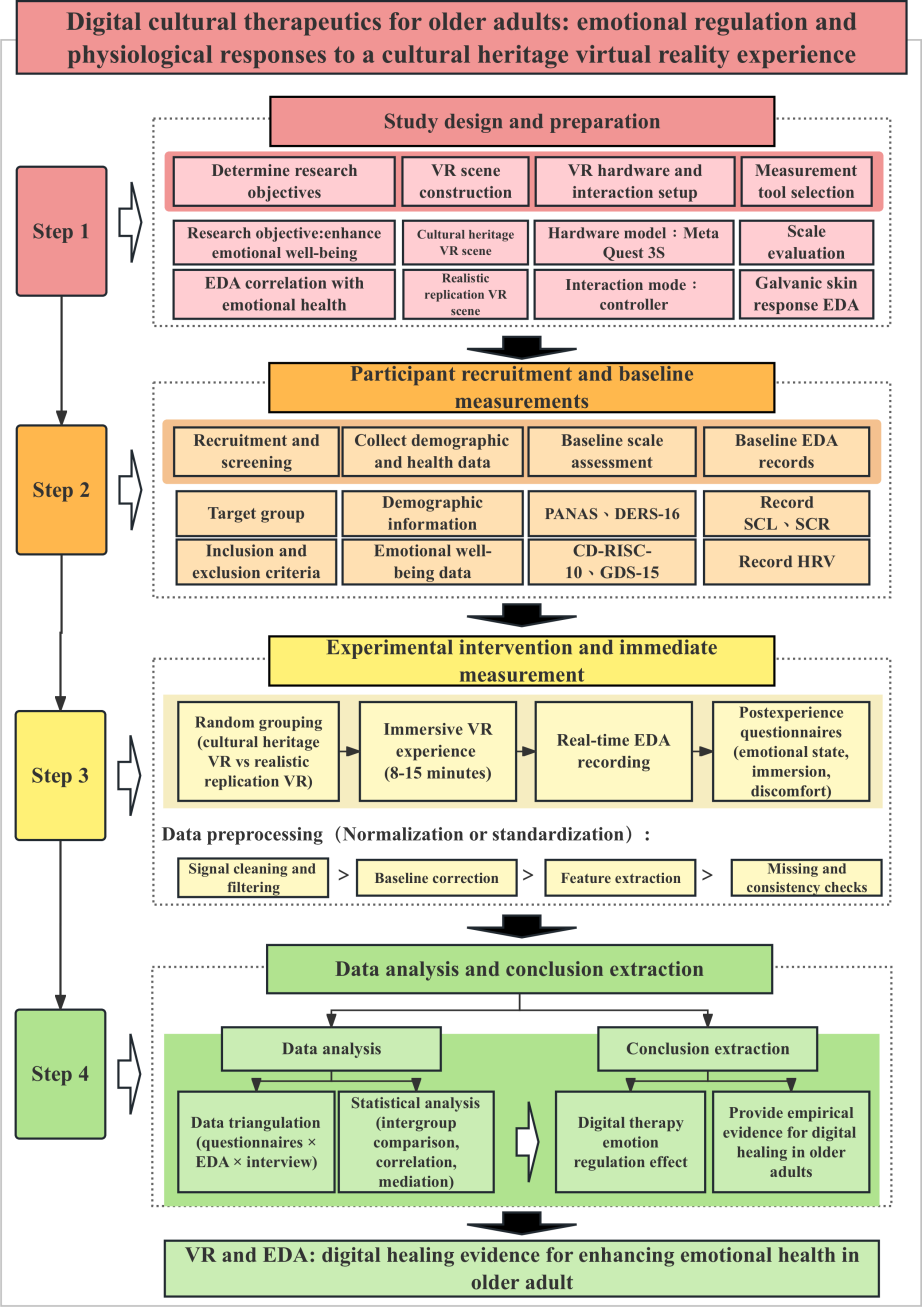
Experimental design of the cultural heritage VR digital therapeutics study. CD-RISC-10: 10-Item Connor-Davidson Resilience Scale; DERS-16: 16-Item Difficulties in Emotion Regulation Scale; EDA: electrodermal activity; GDS-15: 15-Item Geriatric Depression Scale; HRV: heart rate variability; PANAS: Positive and Negative Affect Schedule; SCL: skin conductance level; SCR: skin conductance response; VR: virtual reality.

The study used VR equipment, standardized psychometric questionnaires, and multimodal physiological recording devices to establish an integrated assessment framework combining subjective and objective measures (Figure 1).

#### VR Scene Materials

This study adopted a mixed methods design that combined subjective self-report measures with objective physiological signals to examine the effects of cultural heritage–based VR experiences on emotional health in older adults.

The VR environment was developed based on the Historic Centre of Macao, a United Nations Educational, Scientific and Cultural Organization World Heritage Site, selected due to its rich cultural significance and well-preserved architectural heritage. The inclusion criteria for the VR environment were (1) cultural and historical relevance, (2) visual richness and environmental complexity, and (3) the potential to evoke emotional and cognitive engagement. These criteria were informed by prior research suggesting that immersive and meaningful environments can enhance user engagement and modulate emotional and physiological responses in VR settings [36,37].

**Figure 2.**
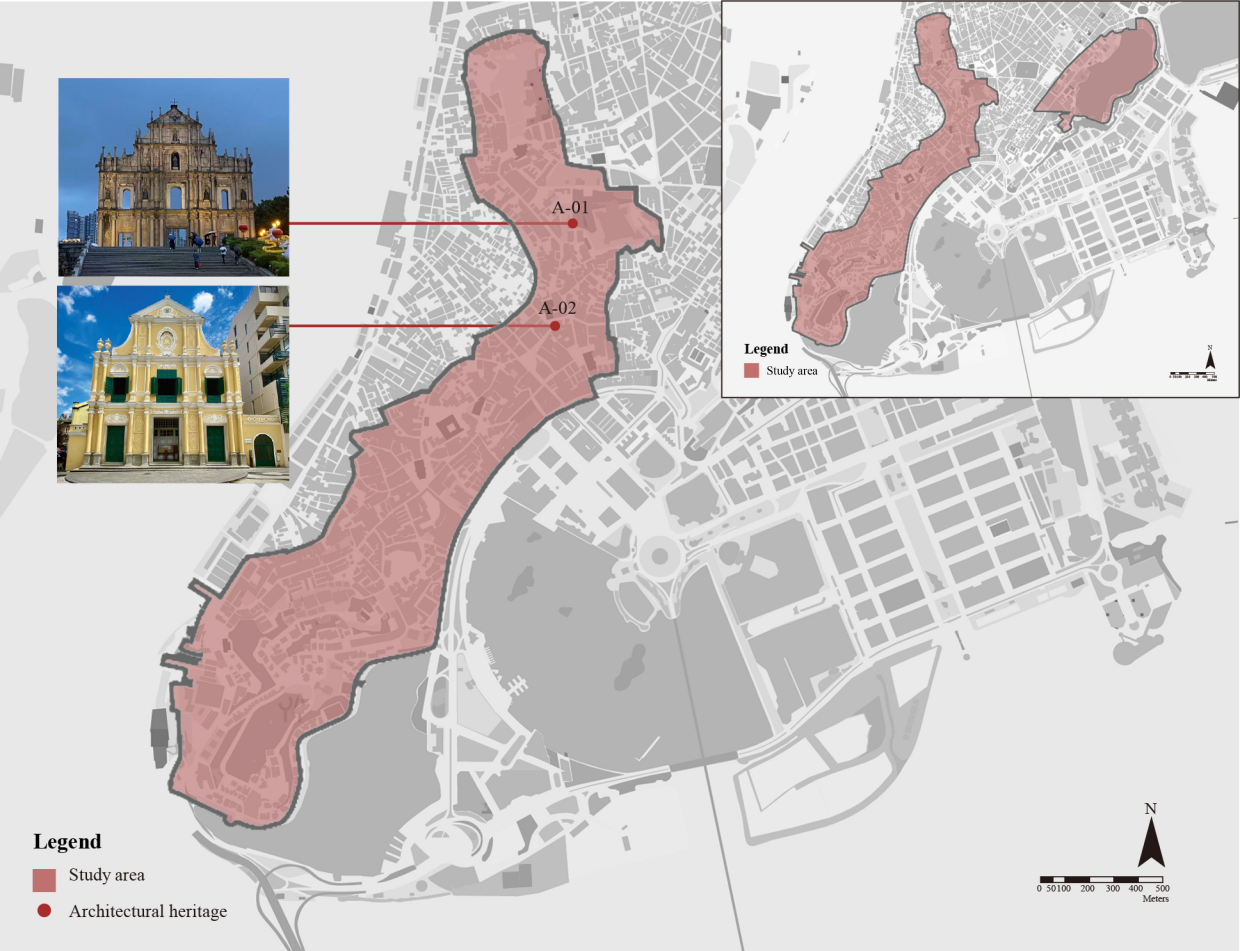
Study area.

The experimental framework comprised 2 parallel VR conditions: a cultural heritage VR scene and a realistic replica VR scene. Both conditions were modeled on the same landmarks—the Ruins of St. Paul’s (A01) and St. Dominic’s Church (A02)—to ensure consistency in spatial content across conditions (Figure 2).

**Figure 3.**
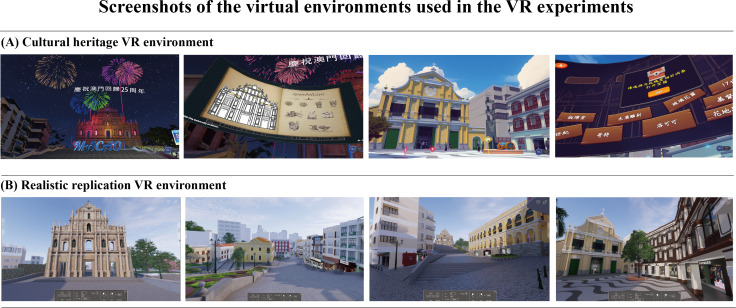
Virtual environment snapshots of the (A) cultural heritage VR and (B) realistic replication VR experimental scenarios. VR: virtual reality.

Participants were randomly assigned to either the cultural heritage VR group or the realistic replica VR group. Prior to the experiment, participants were blinded to their group allocation. The cultural heritage VR scenes incorporated creative enhancements, including visual aesthetic augmentation, soundscape design, light interactive elements, gamified tasks, and embedded cultural information. These features were intended to increase immersion, cultural engagement, and the elicitation of positive emotional responses among older participants. In contrast, the realistic replica VR scenes served as the control condition and were designed to provide a neutral, realistic replication reproduction of the same spaces. These scenes included no background music, no gamified interactions, and no cultural prompts. Visual presentation, auditory input, and interactivity were deliberately kept minimal to control for artistic and experiential factors. Representative snapshots of the virtual environments used in the study are presented in Figure 3.

#### Equipment and Technical Setup

The experimental setup comprised immersive VR hardware and physiological signal acquisition devices. VR experiences were delivered using a head-mounted display (Meta Quest 3/3S), which offers 4K-level resolution, a refresh rate of 90‐120 Hz, and 6 degrees of freedom tracking, ensuring stable and highly immersive visual performance. All VR content was developed by the research team led by PW at the School of Innovative Design, City University of Macau.

To ensure comparability across experimental conditions, the cultural heritage VR and realistic replica VR scenes were matched in spatial structure, viewpoint trajectories, and lighting parameters. The only differences between conditions concerned the presence or absence of artistic enhancements. Specifically, the cultural heritage VR condition incorporated environmental music, narrative voice prompts, cultural knowledge pop-ups, light interactive tasks, and gamified feedback, whereas these elements were deliberately excluded from the realistic replica condition.

Physiological data were collected using the EryLife S2 multimodal sensing device (Wecare Medical Co), which enables synchronized recording of EDA and HRV signals [38]. The sampling frequency for EDA was set at 4 Hz. Given that EDA signals primarily consist of slow-varying tonic and phasic components, relatively low sampling rates have been shown to be sufficient for capturing meaningful skin conductance responses [39]. Photoplethysmography signals were collected at 100 Hz for HRV analysis. This sampling rate is consistent with previous studies demonstrating that ≥100 Hz provides adequate temporal resolution for reliable interbeat interval detection and HRV estimation, in line with established HRV measurement principles [40]. EDA was measured using medical-grade dry electrodes attached to the inner side of the nondominant wrist, ensuring signal stability and wearing comfort for older participants. HRV data were obtained via a photoplethysmography sensor, providing complementary information on sympathetic and parasympathetic nervous system activity.

#### Psychological Measures

To systematically assess baseline emotional health prior to the VR intervention, psychological questionnaires were categorized into three functional domains:

1. Emotional state indicators: Positive and Negative Affect Schedule (PANAS),
2. Emotional burden indicators: Geriatric Depression Scale-15 and University of California, Los Angeles Loneliness Scale-8, and
3. Regulatory trait indicators: Difficulties in Emotion Regulation Scale-16 and Connor-Davidson Resilience Scale-10.

This classification facilitated the distinction between short-term emotional changes (state variables) and relatively stable emotional characteristics (trait variables) in subsequent analyses and supported the examination of potential mechanisms underlying VR intervention effects across different psychological dimensions.

The 20-Item Positive and Negative Affect Schedule [41] was used to assess changes in positive affect (PA) and negative affect (NA) before and after the VR experience. The 16-Item Difficulties in Emotion Regulation Scale (DERS-16) [42] measured individual differences in emotion regulation difficulties, allowing exploration of how regulatory capacity may moderate VR-related emotional benefits. The 10-Item Connor-Davidson Resilience Scale (CD-RISC-10) [43] was administered to evaluate psychological resilience and its potential influence on emotional improvement. The 15-Item Geriatric Depression Scale (GDS-15) [44] served as a screening tool for depressive symptoms in older adults and was used to control for baseline emotional burden. The short version of the University of California, Los Angeles Loneliness Scale [45] assessed perceived social loneliness, enabling further examination of whether immersive VR experiences could alleviate loneliness-related emotional distress.

In addition, demographic variables (eg, living arrangements) and behavioral information (eg, prior exposure to VR) were collected via questionnaire.

All psychological scales used in this study are well-established instruments with demonstrated validity in prior research. To assess internal consistency within the current sample, reliability analyses were conducted by the research team using Cronbach α coefficients in SPSS (IBM Corp). The results indicated satisfactory internal consistency across all measures, with Cronbach α values exceeding the recommended threshold of 0.70.

### Experimental Procedure

#### Overview of the Procedure

Upon arrival at the laboratory, all participants received a standardized explanation of the study procedures and provided written informed consent. Participants then completed baseline psychological assessments, including PANAS, DERS-16, CD-RISC-10, GDS-15, and 8-Item University of California, Los Angeles Loneliness Scale (UCLA-8), to establish initial emotional states, emotional burden, and regulatory and resilience-related traits.

Following the questionnaire assessment, participants underwent a 2-minute resting-state EDA recording in a quiet environment with constant lighting conditions. This baseline recording was used to obtain individual skin conductance level (SCL) and SCR measures. After baseline data collection, participants were randomly assigned by computer algorithm to either the cultural heritage VR group or the realistic replica VR control group.

**Figure 4.**
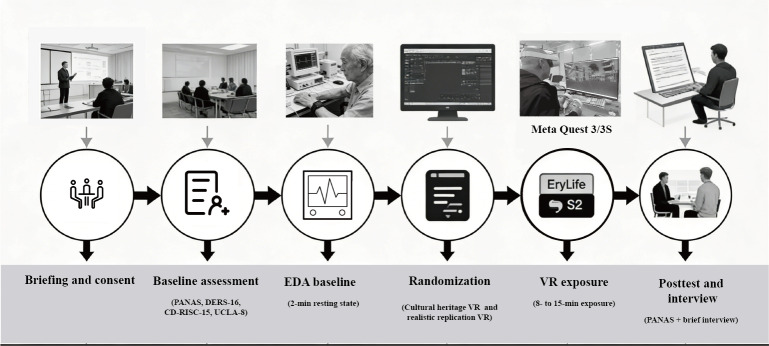
Experimental procedure (illustrative example). CD-RISC-15: 15-Item Geriatric Depression Scale; DERS-16: 16-Item Difficulties in Emotion Regulation Scale; PANAS: Positive and Negative Affect Schedule; UCLA-8: 8-Item University of California, Los Angeles Loneliness Scale; VR: virtual reality.

Participants then engaged in an immersive VR experience lasting approximately 8‐15 minutes using the Meta Quest 3/3S head-mounted display. During the VR session, EDA and heart rate activity were continuously recorded using the EryLife S2 Watch device. Immediately after the VR experience, participants completed the postintervention PANAS assessment and, when appropriate, participated in a brief semistructured interview to supplement subjective experience data (Figure 4).

#### EDA Signal Preprocessing and Feature Extraction

EDA data were continuously collected during the VR experience using the EryLife S2 device and processed through a multistage standardized pipeline. First, the raw signals underwent automated artifact detection and low-pass filtering to remove motion-related noise and signal distortions caused by poor electrode contact. Second, baseline correction was performed using each participant’s resting-state SCL, ensuring comparability across individuals. Subsequently, key EDA features were extracted from the cleaned signals, including SCR frequency, peak amplitude, temporal characteristics, and area under the curve. Finally, *z* score normalization and percentage transformation were applied to optimize data distribution properties and to provide a robust basis for subsequent statistical analyses.

#### RQs and Analytical Strategy

To address the RQs, a controlled experimental design was implemented. Specifically, RQ1 was examined by comparing emotional health outcomes between the cultural heritage VR condition and the realistic replication VR condition using self-report measures. RQ2 was investigated by analyzing physiological emotional responses based on EDA signals recorded during the VR experience. RQ3 was explored by examining the relationships between subjective emotional responses and physiological indicators, with the aim of identifying potential mechanisms underlying emotional health improvements associated with VR exposure.

### Data Processing and Analysis

A multilevel analytical strategy integrating quantitative and qualitative approaches was used to examine the effects of artistic VR experiences on emotional health in older adults and to explore their underlying physiological mechanisms. First, statistical analyses were conducted on questionnaire data, including PANAS, DERS-16, CD-RISC-10, GDS-15, and the 8-Item University of California, Los Angeles Loneliness Scale. Second, to mitigate potential interpretive bias associated with reliance on a single physiological indicator, EDA and HRV signals were jointly analyzed. This multimodal autonomic assessment enabled evaluation of VR-related emotional arousal across multiple dimensions of autonomic nervous system activity. Physiological measures were aggregated at the individual level prior to statistical testing to enhance the robustness and interpretability of the results. Finally, a data triangulation approach was applied to integrate findings from self-report measures, physiological indicators, and semistructured interviews, providing a comprehensive assessment of emotional responses to the VR experience.

To examine changes in emotional states associated with VR exposure, repeated-measures analysis of variance (RM-ANOVA) was conducted on PANAS PA and NA scores, with time (pre- vs postintervention) treated as the within-subject factor and group (cultural heritage VR vs realistic replication VR) as the between-subject factor. Because the within-subject factor consisted of only 2 levels, the assumption of sphericity for RM-ANOVA was inherently satisfied.

## Results

### Physiological Responses

#### Overview

To objectively compare autonomic nervous system responses under different VR conditions in older adults, between-group analyses were conducted using HRV and EDA indicators. For all measures, change scores (Δ=post–pre) were calculated at the individual level and used for statistical comparisons between groups. Continuous variables were expressed as mean (SD). Descriptive analyses were first performed to examine the distributional characteristics of the physiological data. Boxplots were used to visualize the median, IQR, and dispersion of each indicator. Subsequently, nonparametric Mann-Whitney *U* tests were applied to both HRV and EDA measures to assess between-group differences.

#### EDA Result

**Figure 5.**
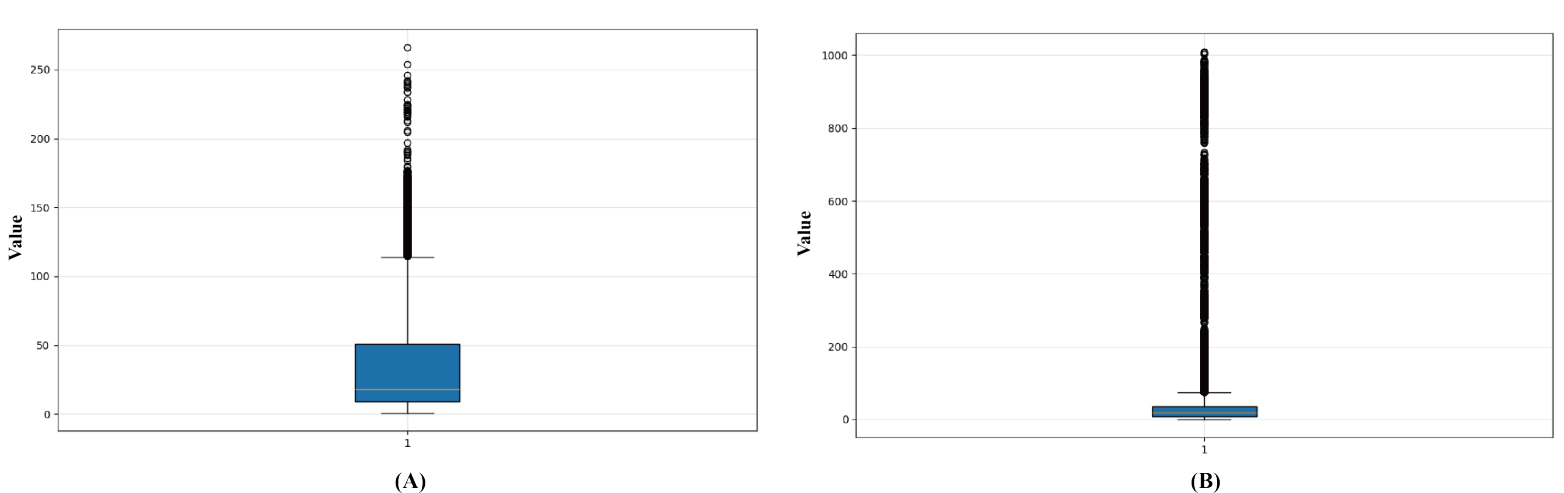
Distributional characteristics of electrodermal activity under different VR conditions: (A) cultural heritage VR and (B) realistic replication VR. VR: virtual reality.

Descriptive analyses revealed clear differences in the distributional properties of EDA between the cultural heritage VR and realistic replica VR conditions. Examination of the boxplots (Figure 5) allowed further differentiation of the sources of these distributional differences.

Under the cultural heritage VR condition, the EDA box (representing the IQR, ie, the middle 50% of observations) was relatively compact, with the median located at a low level (approximately 25), indicating that baseline SCRs were clustered within a narrow range for most participants. However, the upper tail of the distribution was markedly extended and accompanied by several high-value outliers. This pattern indicates that a small subset of participants exhibited SCRs substantially higher than the group norm, thereby considerably increasing overall dispersion.

In the realistic replica VR condition, the EDA box was similarly concentrated in a low-value range, with a relatively low median, suggesting generally stable and homogeneous physiological responses across participants. Although a few high-value outliers were also observed, their number and influence on the overall distribution were limited, resulting in a lower degree of dispersion compared with the cultural heritage VR condition.

Overall, the primary differences between the 2 VR conditions lay in distributional dispersion and upper-tail structure rather than in a pronounced shift in central tendency. These findings suggest that cultural heritage VR experiences are more likely to elicit pronounced interindividual variability in autonomic responses, whereas realistic replica VR experiences are associated with more uniform and stable EDA response patterns.

The between-group comparisons of EDA indicators are presented in Table 2. No statistically significant differences were observed between the 2 VR conditions across all EDA measures, including mean EDA (*P*=.68; Cohen *d*=−0.16), SCL (*P*=.66; Cohen *d*=−0.16), SCR amplitude (*P*=.80; Cohen *d*=−0.09), and SCR peak rate (*P*=.87; Cohen *d*=0.06). All effect sizes were small (|*d*|<0.20), indicating limited practical differences in EDA responses between the 2 conditions.

**Table 2.** Group comparisons of electrodermal activity (EDA) indicators.

Signal source and feature	Cultural heritage VR^a^ (n=25), mean (SD)	Realistic replication VR (n=24), mean (SD)	*P* value	Cohen *d*
EDA				
Mean EDA	1.58 (4.09)	2.61 (8.75)	.68	−0.16
SCL^b^	1.52 (4.12)	2.61 (8.77)	.66	−0.16
SCR^c^ amplitude	−0.04 (0.44)	0.02 (0.81)	.80	−0.09
SCR peak rate	−0.29 (4.33)	−0.56 (4.67)	.87	0.06

a VR: virtual reality.

b SCL: skin conductance level.

c SCR: skin conductance response.

**Figure 6.**
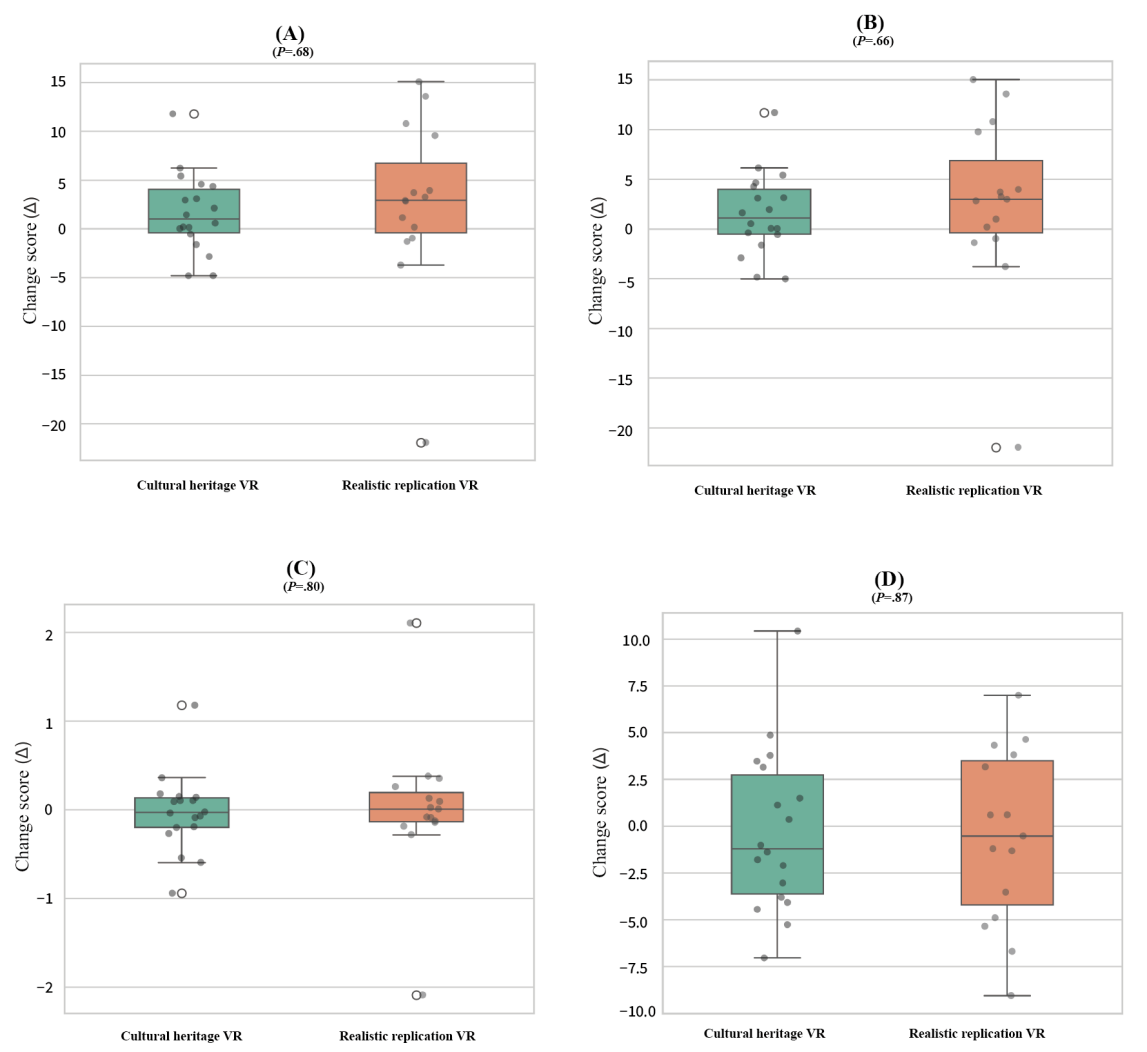
Changes in EDA indicators across VR conditions. (A) Mean EDA, (B) SCL, (C) SCR amplitude, and (D) SCR peak rate. EDA: electrodermal activity; HRV: heart rate variability; SCL: skin conductance level; SCR: skin conductance response; VR: virtual reality.

Distributional analyses (Figure 6) further showed substantial overlap in medians and IQRs across groups, with no clear separation between conditions. Although several indicators exhibited occasional extreme values, these variations were primarily observed at the individual level and did not translate into consistent group-level effects.

From a physiological perspective, SCL reflects tonic sympathetic arousal, whereas SCR-related measures (amplitude and peak rate) capture phasic, event-related responses. These findings suggest that, under the present experimental conditions, EDA indicators did not demonstrate consistent group-level differentiation, potentially reflecting high interindividual variability and the complex nature of emotional arousal in immersive environments.

#### HRV Result

The between-group comparisons of HRV indicators are presented in Table 3. The results showed that a significant difference was observed for the mean HRV-derived value between the 2 VR conditions (*P*=.01; Cohen *d*=0.91), with higher change scores in the cultural heritage VR group (mean 21.48, SD 30.39) compared to the realistic replication VR group (mean −2.69, SD 21.03).

**Table 3.** Group comparisons of heart rate variability (HRV) indicators.

Signal source and feature	Cultural heritage VR^a^ (n=25), mean (SD)	Realistic replication VR (n=24), mean (SD)	*P* value	Cohen *d*
HRV				
Mean NN^b^ interval	21.48 (30.39)	−2.69 (21.03)	.01	0.91
SDNN^c^	0.71 (11.26)	12.40 (20.00)	.06	−0.74
RMSSD^d^	−0.96 (20.70)	3.52 (14.89)	.48	−0.24

a VR: virtual reality.

b NN: normal-to-normal.

c SDNN: standard deviation of NN.

d RMSSD: root-mean-square of successive difference.

In contrast, conventional time-domain HRV indices, including the standard deviation of NN intervals (SDNN) and the root-mean-square of successive differences (RMSSD), did not show statistically significant differences between groups. Although SDNN exhibited a numerical difference (*P*=.06; Cohen *d*=−0.74), it did not reach statistical significance. Similarly, no significant difference was found for RMSSD (*P*=.48; Cohen *d*=−0.24).

**Figure 7.**
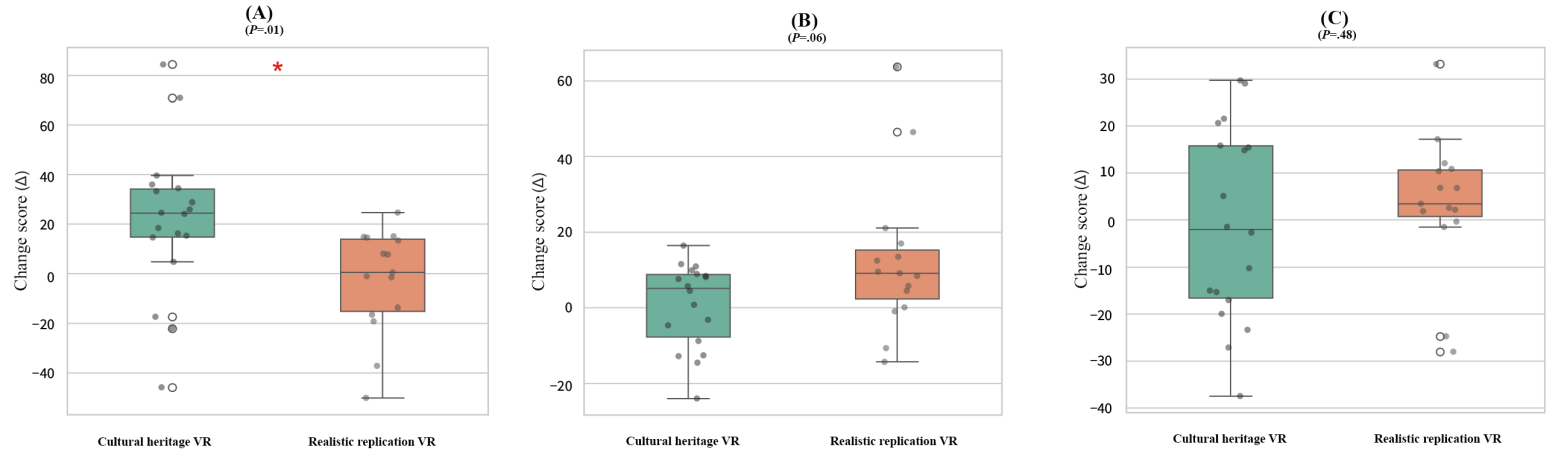
Changes in heart rate variability indicators across VR conditions. (A) Mean NN interval, (B) SDNN, and (C) RMSSD. NN: normal-to-normal; RMSSD: root-mean-square of successive differences; SDNN: standard deviation of NN; VR: virtual reality.

Distributional analyses (Figure 7) indicated that the mean normal-to-normal (NN) interval (mean RR interval) tended to be higher under the cultural heritage VR condition, whereas substantial overlap was observed between groups for SDNN and RMSSD. Overall, the between-group difference was primarily reflected in the mean NN interval, while no consistent pattern was observed across standard time-domain HRV measures.

From a physiological perspective, RMSSD is generally considered to reflect parasympathetic activity, whereas SDNN represents overall HRV. However, as these conventional indices did not show significant differences, the physiological interpretation of the observed group difference should be approached with caution. The findings suggest that the effects of different VR conditions on autonomic regulation may be limited or influenced by substantial interindividual variability.

### Subjective Emotional Outcomes and Psychological Measures

#### Baseline Sample Characteristics and Between-Group Comparability

To ensure comparability between the cultural heritage VR group and the realistic replica VR group prior to intervention, chi-square tests were conducted on participants’ demographic and health-related background variables. A total of 12 indicators were examined, including sex, age group, educational attainment, marital status, living arrangement, occupational background, economic status, visual and auditory conditions, sleep quality, experience with smart devices, and prior exposure to VR or digital therapeutic applications (Table 4).

No statistically significant differences were observed between the 2 groups for any of the variables examined (*χ*^2^ tests; all *P*>.05). These results indicate a high degree of baseline equivalence between groups in terms of sociodemographic structure, health status, and technology-related experience, thereby minimizing potential confounding effects attributable to sample composition and providing a reliable baseline for subsequent analyses of emotional and physiological outcomes.

**Table 4.** Results of the chi-square analysis.

Item and response options	Group		Chi-square (*df*)	*P* value
	Cultural heritage VR^a^, n (%)	Realistic replication VR, n (%)		
Sex			0.2 (1)	.67
Female	14 (56)	12 (50)		
Male	11 (44)	12 (50)		
Age group (years)			0.9 (3)	.84
60‐64	11 (44)	8 (33)		
65‐69	5 (20)	5 (21)		
70‐74	5 (20)	5 (21)		
75 and above	4 (16)	6 (25)		
Educational attainment			1.7 (2)	.44
Junior college (associate degree)	5 (20)	4 (17)		
Bachelor's degree	5 (20)	2 (8)		
High school or below	15 (60)	18 (75)		
Marital status			2.0 (2)	.37
Widowed	1 (4)	0 (0)		
Married	23 (92)	24 (100)		
Unmarried	1 (4)	0 (0)		
Living arrangement			4.0 (3)	.26
Living with children	11 (44)	14 (58)		
Living with spouse	8 (32)	5 (21)		
Living alone	0 (0)	2 (8)		
Other	6 (24)	3 (13)		
Current occupational status			3.1 (3)	.38
Other	2 (8)	2 (8)		
Retired	20 (80)	22 (92)		
Self-employed or freelance	2 (8)	0 (0)		
Re-employed after retirement	1 (4)	0 (0)		
Economic status			5.3 (3)	.15
Generally sufficient	13 (52)	19 (79)		
Very difficult	1 (4)	0 (0)		
Somewhat difficult	2 (8)	0 (0)		
Relatively comfortable	9 (36)	5 (21)		
Visual condition			2.2 (2)	.33
Slightly blurred but adequate for daily activities without glasses	6 (24)	3 (12)		
Normal vision without glasses	18 (72)	21 (88)		
Requires glasses for normal	1 (4)	0 (0)		
Hearing condition			0.0 (1)	.98
Does not require a hearing aid but needs louder or repeated speech to hear clearly	1 (4)	1 (4)		
Normal hearing without a hearing aid	24 (96)	23 (96)		
Sleep quality			1.8 (3)	.62
Fair	7 (28)	5 (21)		
Very good	7 (28)	7 (29)		
Poor	3 (12)	1 (4)		
Average	8 (32)	11 (46)		
Use of smart devices (smartphone or tablet)			0.0 (1)	.98
No	1 (4)	1 (4)		
Yes	24 (96)	23 (96)		
Prior exposure to VR or digital therapeutic products			1.6 (1)	.20
No	23 (92)	19 (79)		
Yes	2 (8)	5 (21)		

a VR: virtual reality.

#### Between-Group Differences in Baseline Psychological Traits

Prior to the VR intervention, 2-tailed independent-samples *t* test were performed to compare baseline psychological trait measures between groups, including emotion regulation difficulties (DERS-16), psychological resilience (CD-RISC-10), and depressive symptoms (GDS-15). No significant between-group differences were detected for emotion regulation difficulties (*t*_47_=−1.08; *P*=.29), psychological resilience (*t*_25.538_=1.869; *P*=.07), or depressive symptoms (*t*_31.212_=−0.688; *P*=.49; Table 5). These findings further confirm baseline homogeneity between the 2 groups at the psychological trait level, establishing an appropriate foundation for evaluating VR-induced emotional changes.

**Table 5.** Results of 2-tailed independent-samples *t* tests.

Variable	Group		*t* test (*df*)	*P* value
	Cultural heritage VR^a^ experience (n=25), mean (SD)	Realistic replication VR experience (n=24), mean (SD)		
Emotion regulation difficulties	39.00 (8.48)	41.67 (8.81)	−1.08 (47)	.29
Psychological resilience	39.48 (1.19)	38.13 (3.35)	1.869 (25.538)	.07
Depression	23.44 (0.77)	23.71 (1.76)	−0.688 (31.212)	.49

a VR: virtual reality.

#### Effects of VR Intervention on PA and NA

To examine the effects of VR exposure on emotional state, RM-ANOVA was conducted on PANAS PA and NA scores, with time (pre- vs postintervention) as the within-subject factor and group (cultural heritage VR vs realistic replica VR) as the between-subject factor.

Descriptive statistics are presented in Table 6. At baseline, PA and NA scores were comparable between the 2 groups. Following the intervention, PA increased and NA decreased in both groups, with more pronounced changes observed in the cultural heritage VR group.

For PA, RM-ANOVA revealed a significant main effect of time (*F*_1,47_=211.20; *P*<.001; η^2^=0.818), indicating an overall increase following VR exposure. A significant main effect of group (*F*_1,47_=5.67; *P*=.02; η^2^=0.108) and a significant time×group interaction (*F*_1,47_=12.47; *P*=.001; η^2^=0.210) were also observed (Table 7).

**Table 6.** Descriptive statistics of positive affect (PA) and negative affect (NA) scores before and after the virtual reality (VR) intervention.

Measure and time	Cultural heritage VR experience (n=25), mean (SD)	Realistic replication VR experience (n=24), mean (SD)
PA		
Preintervention	30.96 (1.54)	30.79 (2.86)
Postintervention	43.48 (4.36)	38.42 (7.34)
NA		
Preintervention	19.00 (2.24)	21.38 (6.44)
Postintervention	10.32 (1.11)	17.46 (6.98)

**Table 7.** Repeated-measures ANOVA results.

Measure and effect	Type III sum of squares	*df*	Mean square	*F* test (*df=*1, 47)	*P* value	Effect size (η^2^)
PA^a^						
Time	2484.619	1	2484.619	211.196	<.001	0.818
Group	167.573	1	167.573	5.674	.02	0.108
Time×group	146.7	1	146.7	12.47	.001	0.210
NA^b^						
Time	971.486	1	971.486	118.709	<.001	0.716
Group	554.103	1	554.103	14.151	<.001	0.231
Time×group	138.914	1	138.914	16.974	<.001	0.265

a PA: positive affect.

b NA: negative affect.

For NA, a similar pattern was observed. RM-ANOVA showed a significant main effect of time (*F*_1,47_=118.71; *P*<.001; η^2^=0.716), along with significant main effects of group (*F*_1,47_=14.15; *P*<.001; η^2^=0.231) and a significant time× group interaction (*F*_1,47_=16.97; *P*<.001; η^2^=0.265; Table 7).

Simple effects analyses (Tables8  9) revealed a consistent pattern of significant time effects and differential group effects over time. At baseline, no significant between-group differences were observed in either PA or NA. Following the intervention, PA increased significantly in both the cultural heritage VR experience group and the realistic replication VR experience group; however, the increase was significantly greater in the cultural heritage VR group, resulting in higher postintervention PA compared with the control condition. Conversely, NA decreased significantly in both groups across time, with a significantly larger reduction observed in the cultural heritage VR group, which also exhibited significantly lower postintervention NA relative to the realistic replication VR group.

**Table 8.** Within-group simple effects analysis of positive affect (PA) and negative affect (NA) across time points^a^.

Measure and group	Comparison	Mean difference (SE)	*P* value
PA			
Cultural heritage VR^b^ experience	Pre versus post	−12.520 (0.970)	<.001
Realistic replication VR experience	Pre versus post	−7.625 (0.990)	<.001
NA			
Cultural heritage VR experience	Pre versus post	8.680 (0.809)	<.001
Realistic replication VR experience	Pre versus post	3.917 (0.826)	<.001

a Time 1=preintervention; time 2=postintervention.

b VR: virtual reality.

**Table 9.** Between-group simple effects analysis of positive affect (PA) and negative affect (NA) at each time point^a^.

Measure and time	Comparison	Mean difference (SE)	*P* value
PA			
Pre	Cultural heritage VR^b^ experience versus realistic replication VR experience	0.168 (0.652)	.80
Post	Cultural heritage VR experience versus realistic replication VR experience	5.063 (1.717)	.005
NA			
Pre	Cultural heritage VR experience versus realistic replication VR experience	−2.375 (1.366)	.09
Post	Cultural heritage VR experience versus realistic replication VR experience	−7.138 (1.414)	<.001

a Time 1=preintervention; time 2=postintervention*.*

b VR: virtual reality.

#### Associations Between Psychological Traits and Emotional Changes

To further explore individual differences in emotional responses to VR, Pearson correlation analyses were conducted to examine associations among emotion regulation difficulties, psychological resilience, depressive symptoms, and emotional state before and after the intervention (Table 10).

**Table 10.** Pearson correlation analysis between emotion regulation difficulty, depression, and emotional indicators before and after the intervention.

Variable and statistic	Difficulties in emotion regulation	Psychological resilience	Depression	Preintervention PA^a^	Preintervention NA^b^	Postintervention PA	Postintervention NA
Difficulties in emotion regulation							
*r*	1	−0.530	0.526	−0.525	0.638	−0.625	0.425
*P* value	—^c^	<.001	<.001	<.001	<.001	<.001	.002
Psychological resilience							
*r*	−0.530	1	−0.649	0.600	−0.754	0.770	−0.477
*P* value	<.001	—	<.001	<.001	<.001	<.001	<.001
Depression							
*r*	0.526	−0.649	1	−0.761	0.754	−0.661	0.441
*P* value	<.001	<.001	—	<.001	<.001	<.001	.002
Preintervention PA							
*r*	−0.525	0.600	−0.761	1	−0.699	0.610	−0.327
*P* value	<.001	<.001	<.001	—	<.001	<.001	.02
Preintervention NA							
*r*	0.638	−0.754	0.754	−0.699	1	−0.736	0.657
*P* value	<.001	<.001	<.001	<.001	—	<.001	<.001
Postintervention PA							
*r*	−0.625	0.770	−0.661	0.610	−0.736	1	−0.609
*P* value	<.001	<.001	<.001	<.001	<.001	—	<.001
Postintervention NA							
*r*	0.425	−0.477	0.441	−0.327	0.657	−0.609	1
*P* value	.002	<.001	.002	.02	<.001	<.001	—

a PA: positive affect.

b NA: negative affect.

c Not available.

Emotion regulation difficulties were significantly negatively correlated with psychological resilience (*r=*−0.530; *P*<.01) and positively correlated with depressive symptoms (*r*=0.526; *P*<.01). In addition, higher emotion regulation difficulties were associated with lower PA and higher NA both before and after the VR experience, indicating greater emotional burden among individuals with poorer regulation capacity.

Psychological resilience showed the opposite pattern, exhibiting significant positive correlations with PA and significant negative correlations with NA and depressive symptoms. These findings suggest that resilience may serve a buffering and facilitative role in VR-based emotional interventions.

#### Convergence Between Subjective Emotional Outcomes and EDA Responses

Comparative analysis of subjective emotional changes and EDA responses revealed consistent patterns across modalities. Relative to the realistic replica VR condition, participants exposed to cultural heritage VR exhibited higher and more sustained levels of skin conductance during the experience, alongside larger increases in PA and more pronounced reductions in NA on self-report measures.

This convergence between subjective emotional outcomes and autonomic physiological responses indicates coherent emotion-related effects across measurement modalities under different VR content conditions, providing an empirical basis for subsequent multimodal interpretations of VR-induced emotional regulation effects.

## Discussion

### Convergence of Subjective and Physiological Evidence and the Emotional Regulation Potential of Cultural Heritage VR

This study provides an exploratory evaluation of the role of cultural heritage VR in promoting emotional health among older adults by integrating subjective emotional measures with physiological indicators. The findings indicate that, compared with the visually neutral realistic replication VR condition, cultural heritage VR was associated with higher PA and lower NA. At the physiological level, only the mean NN interval showed a statistically significant between-group difference. In contrast, no significant differences were observed in EDA measures (all *P*>.05). However, EDA measures showed substantial interindividual variability in their distribution patterns. It should be noted that the mean NN interval reflects overall cardiac cycle length rather than conventional HRV variability indices.

From the perspective of emotional arousal theory, changes in EDA are widely regarded as sensitive indicators of sympathetic nervous system activation and are closely linked to emotional engagement and attentional resource allocation [39,46,47]. The cultural heritage VR condition in this study incorporated enhanced visual aesthetics, soundscape design, narrative cues, and light interactive elements. These features may have facilitated affective engagement and sustained attention among older participants. However, this potential emotional arousal did not translate into stable group-level differences in EDA measures. Instead, it was reflected as variability at the individual level. Notably, although the physiological findings were limited, subjective emotional improvements showed a clearer pattern of differentiation between the 2 conditions. The discrepancy between subjective emotional improvement and physiological responses suggests a potential dissociation between perceived experience and autonomic activation.

This dissociation may be explained by several mechanisms. First, EDA primarily reflects sympathetic arousal. It may be less sensitive to changes in emotional valence when arousal levels remain moderate. In this study, the cultural heritage VR condition appeared to enhance PA without inducing strong physiological arousal, resulting in limited differentiation in EDA responses at the group level. Second, emotional engagement in VR environments may operate through cognitive-affective pathways, such as meaning-making and narrative immersion, which are more strongly reflected in subjective reports than in autonomic activation. Third, substantial interindividual variability in EDA responses may have obscured potential group-level effects. This variability may be particularly pronounced in older populations, where physiological responsiveness differs considerably.

### Comparison With Previous Research: Cultural Heritage VR, Digital Therapeutics, and Emotional Health in Older Adults

Previous studies have widely reported that VR-based interventions can improve negative emotional states, enhance subjective well-being, and increase psychological engagement among older adults. These applications have primarily focused on rehabilitation training, cognitive stimulation, and social participation [11,48,49]. In such studies, emotional improvements are often attributed to immersion, novelty, and heightened attentional engagement afforded by VR technology [50,51].

However, much of the existing literature conceptualizes VR primarily as a technological medium, with limited systematic discussion of how artistic expression and cultural meaning embedded in virtual content contribute to emotional regulation [38]. In contrast, this study explicitly distinguishes between cultural heritage VR and realistic replica VR. Empirical evidence demonstrates that different types of virtual content do not elicit equivalent emotional responses. Emotional changes in older participants were not driven solely by VR technology itself but were more strongly influenced by artistic expression, cultural narrative structures, and experiential guidance embedded within the virtual environment. This finding extends existing VR intervention research by highlighting the emotional consequences of content differentiation.

Recent studies have begun incorporating music, narrative, or aesthetic elements into VR interventions and have reported positive outcomes at the subjective level. Nonetheless, most rely heavily on self-report measures and lack objective physiological validation [52-54]. By synchronously collecting EDA data, this study provides a complementary perspective to the existing literature. However, in contrast to prior studies reporting significant physiological changes, no significant between-group differences were observed in EDA indicators in this study. This discrepancy may be attributable to differences in experimental design and stimulus characteristics. Prior studies reporting significant physiological changes often used high-intensity or event-based stimuli, whereas this study used a more continuous and immersive experiential paradigm. Such differences may lead to distinct patterns of autonomic activation. Continuous experiences may elicit more subtle and distributed physiological responses. These responses may not be readily captured by conventional EDA metrics. This finding suggests that the emotional effects of VR interventions may not necessarily be accompanied by pronounced increases in physiological arousal, but may instead be more prominently reflected through subjective experiential pathways.

Nevertheless, distributional patterns indicate that a subset of participants in the cultural heritage VR condition exhibited relatively higher physiological responses, suggesting a greater potential for emotional activation at the individual level. Accordingly, the emotional effects of VR interventions appear to be partially individual-dependent, with physiological manifestations likely influenced by multiple factors, including individual differences and the degree of engagement with the virtual environment. These results support a shift in digital therapeutics research from a technology-centered effectiveness perspective toward a content- and experience-driven explanatory framework.

### Mechanistic Pathways and Design Implications: A Conceptual Framework for Digital Therapeutics

Based on the integrated analysis of subjective emotional assessments and EDA data, this study proposes a conceptual pathway to explain the emotional effects of cultural heritage VR. The pathway emphasizes that culturally meaningful artistic experiences are associated with elevated emotional arousal, which in turn corresponds to changes in emotional states among older adults. It should be noted that this pathway is interpretative and hypothesis-generating rather than a fully validated causal model.

At the experiential level, cultural heritage VR may foster sustained emotional engagement and meaning construction through aesthetic visual language, soundscape design, narrative cues, and light interactive tasks. These features transform virtual environments from purely observable spaces into culturally situated emotional contexts, providing conditions conducive to emotional activation. At the physiological level, higher levels of affective engagement and sustained attentional states may be reflected in autonomic activation, as indicated by electrodermal responses in a subset of individuals. However, this effect does not manifest as a consistent difference at the group level. This pattern suggests that emotional processing in VR may involve partially independent pathways for subjective experience and physiological activation. Affective engagement and meaning construction may enhance perceived emotional states. However, their translation into measurable autonomic responses may depend on factors such as stimulus intensity, individual sensitivity, and attentional dynamics. Unlike stress-induced or high-intensity stimulation responses, the observed EDA changes are more consistent with moderate, regulatable emotional arousal and co-occur with increased PA and reduced NA. This suggests that physiological arousal elicited by cultural heritage VR is more indicative of emotional participation and meaning experience rather than nonspecific sensory stimulation.

From the perspective of digital therapeutics and design practice, this conceptual pathway carries important implications. The findings underscore that content design in VR interventions is not ancillary but may constitute a primary condition for emotional effects to emerge. Moreover, incorporating physiological measures such as EDA into experience evaluation frameworks can address limitations inherent in exclusive reliance on self-report data and provide a more objective, verifiable basis for emotional health assessment in older adults. Future studies may further refine this pathway by introducing psychological constructs such as immersion and presence to test mediating mechanisms with greater precision. This pathway further suggests that emotional effects may depend on the dynamic coupling among contextual meaning, affective engagement, and physiological responses, rather than being driven by stimulus intensity alone.

### Limitations and Future Research Directions

Despite its systematic design and methodological integration, this study has several limitations that warrant further investigation. First, the sample size was relatively modest, and participants were recruited from community settings within a limited geographic region. This may introduce homogeneity in cultural background and technology acceptance, thereby constraining the generalizability of the findings. Future research should incorporate multiregional and cross-cultural samples to assess the broader applicability of cultural heritage VR–based emotional interventions.

Second, the study primarily examined the immediate or short-term emotional effects of a single VR exposure. Long-term outcomes and the stability of emotional benefits remain unclear and should be explored through longitudinal designs with follow-up assessments. Third, although EDA was used as a sensitive indicator of emotional arousal, it cannot fully capture the complexity of autonomic nervous system responses. Future work may integrate additional physiological measures, such as electroencephalography or eye-tracking, to construct a more comprehensive multimodal analytical framework.

Furthermore, while the study emphasizes the emotional value of artistic design in cultural heritage VR, the experimental conditions incorporated multiple experiential elements simultaneously. The cultural heritage VR environment included background music, narrative voice prompts, interactive tasks, and cultural information pop-ups, whereas the photorealistic VR condition provided a comparatively minimal stimulus environment. As a result, the observed emotional differences may not be attributable solely to the cultural heritage content itself. Additional factors such as auditory stimulation, increased interactivity, task engagement, or novelty effects may also have contributed to the observed outcomes. This study therefore cannot fully disentangle the independent and interactive effects of these design components. Future research should adopt more controlled experimental paradigms—such as factorial or modular designs—in which specific elements (eg, auditory cues, interaction levels, narrative guidance, or cultural storytelling) are systematically manipulated in order to clarify their relative contributions to emotional responses.

In addition, this study did not directly assess immersion or sense of presence. Future research could incorporate validated presence scales or structural equation modeling approaches to more precisely examine the mediating role of emotional arousal within this process. Overall, this study provides preliminary but robust empirical support for the application of artistic VR in promoting emotional health among older adults. It also offers a methodological and theoretical foundation for future research at the intersection of design studies, environmental psychology, and digital therapeutics.

### Conclusions

This study aimed to examine the effects of cultural heritage–based VR on emotional health in older adults, in comparison with a realistic replication VR condition. In addition, the study explored the relationships between subjective emotional responses, physiological indicators (HRV and EDA), and individual psychological characteristics.

The findings indicate that, compared with the realistic replication VR condition, cultural heritage VR significantly enhanced PA and reduced NA. These results support the hypothesis that culturally meaningful and artistically enriched VR environments can more effectively promote emotional well-being in older adults.

At the physiological level, a significant between-group difference was observed only for the mean NN interval, whereas no statistically significant differences were found in EDA measures. Furthermore, physiological indicators did not demonstrate fully consistent relationships with subjective emotional changes, suggesting that physiological responses to VR may be more variable and influenced by individual differences.

In addition, emotion regulation–related traits and psychological resilience were found to be associated with emotional outcomes, highlighting the importance of individual psychological characteristics in shaping responses to VR interventions. These findings provide preliminary support for a conceptual pathway linking immersive experience to emotional health, although the underlying mechanisms require further empirical investigation.

This study contributes to the field in 3 main ways. First, it provides empirical evidence supporting the emotional benefits of cultural heritage VR as a potential digital intervention for older adults. Second, it demonstrates the value of integrating subjective measures with physiological indicators to achieve a more comprehensive assessment of emotional responses. Third, it offers a preliminary framework for understanding the mechanisms underlying VR-induced emotional change.

From a practical perspective, the findings inform the design of culturally meaningful and emotion-oriented VR applications for aging populations. Future research should examine the long-term effects of such interventions in larger and more diverse samples and further explore the generalizability of artistic VR approaches across different cultural contexts.

## Abbreviations

CD-RISC-10: 10-Item Connor-Davidson Resilience Scale
DERS-16: 16-Item Difficulties in Emotion Regulation Scale
EDA: electrodermal activity
GDS-15: 15-Item Geriatric Depression Scale
HRV: heart rate variability
NA: negative affect
NN: normal-to-normal
PA: positive affect
PANAS: Positive and Negative Affect Schedule
RM-ANOVA: repeated-measures analysis of variance
RMSSD: root-mean-square of successive difference
RQ: research question
SCL: skin conductance level
SCR: skin conductance response
SDNN: standard deviation of NN
VR: virtual reality
